# The 40bp Indel Polymorphism rs150550023 in the *MDM2* Promoter is Associated with Intriguing Shifts in Gene Expression in the p53-MDM2 Regulatory Hub

**DOI:** 10.3390/cancers12113363

**Published:** 2020-11-13

**Authors:** Heidi Miedl, Bianca Dietrich, Klaus Kaserer, Martin Schreiber

**Affiliations:** 1 Department of Obstetrics & Gynecology, Medical University of Vienna, 1090 Vienna, Austria; heidi.miedl@muv.ac.at (H.M.); bianca.dietrich@muv.ac.at (B.D.); 2 Labor Kaserer, Salzer and Beer, 1030 Vienna, Austria; office@labor-kaserer.at; 3 Comprehensive Cancer Center, Medical University of Vienna, 1090 Vienna, Austria

**Keywords:** breast cancer, MDM2 (human homolog of mouse double minute 2), rs150550023, rs3730485, Indel polymorphism, mRNA expression, TP53, age at onset, prognosis, tumor tissue

## Abstract

**Simple Summary:**

Many naturally occurring variants in the human DNA sequence have an influence on cancer risk, and most of them are located outside the parts of the genome that code for proteins. Presumably, they have an effect on the amount of messenger RNA and protein that is made from genes in their neighborhood. We analyzed one such DNA sequence variant termed rs150550023, which is near a gene termed *MDM2* (human homolog of mouse double minute 2). The main function of *MDM2* is to negatively regulate another gene termed p53, which is a very important tumor suppressor, i.e., it counteracts cancer. We studied the DNA sequence variant rs150550023 by comparing it in 407 female patients with breast cancer and 254 females without cancer. We found no evidence that rs150550023 plays an important role in the risk of breast cancer, the average age at which patients get breast cancer, how many patients develop breast cancer metastases, or how many patients die of breast cancer. However, we found evidence that rs150550023 may work together with another DNA sequence variant within the *MDM2* gene termed SNP309. We also analyzed the tumor tissue of ≈100 breast cancer patients of this study after it had been surgically removed. We measured the amount of messenger RNA (mRNA) which is made from the genes *MDM2*, p53, and three other genes (termed p21, *BAX*, and *PERP*) for which it is known that p53 has an influence on the amount of their mRNA being made. We found that rs150550023 indeed has an influence on the amount of mRNA made from some of these genes. However, since MDM2 is a negative regulator of p53, it is likely that many of these alterations cancel each other out if both p53 and MDM2 is produced at higher levels.

**Abstract:**

Most low-penetrance genetic risk factors for cancer are located in noncoding regions, presumably altering the regulation of neighboring genes. The poorly characterized Indel polymorphism rs150550023 (rs3730485; del1518) in the promoter of *MDM2* (human homolog of mouse double minute 2) is a biologically plausible candidate genetic risk factor, which might influence the expression of *MDM2*, a key negative regulator of the central tumor suppressor p53. Here, we genotyped rs150550023 in a Central European hospital-based case–control study of 407 breast cancer patients and 254 female controls. mRNA levels of MDM2, p53, and the p53 target genes p21, BAX, and PERP were quantified with qRT-PCR, and p53 protein was assessed with immune histochemistry in ≈100 primary breast tumors with ascertained rs150550023 genotype. We found no evidence for an association of rs150550023 with the risk, age at onset, or prognosis of breast cancer. A possible synergism was observed with SNP309 in promoter P2 of *MDM2*. Mean mRNA levels of MDM2, p53, p21, and BAX were ≈1.5–3 fold elevated in *TP53* wildtype tumors with the minor homozygous Del/Del genotype. However, systematic shifts in p53 protein levels or mutation rates were not observed, suggesting that the elevated p53 mRNA levels are due to regulatory feedback loops that compensate for the effects of rs150550023 on MDM2 expression.

## 1. Introduction

The tumor suppressor gene *TP53* is the most commonly mutated gene in human cancer [1,2]. Multiple and diverse stress signals activate p53, mostly by interfering with the negative regulatory activity of MDM2 towards p53 [3,4]. Once activated, p53 initiates a similarly diverse set of responses, resulting in either cell death or repair of the stress-induced damages and maintenance of cellular homeostasis [3,4,5,6,7]. p53 is a transcription factor, and one of its target genes is *MDM2*, an E3 ubiquitin ligase and key negative regulator of p53 [8,9,10,11]. MDM2 targets p53 for proteasomal degradation, and also controls its transcriptional activity, nuclear localization, and rate of translation [10,11,12]. Thus, by inducing the expression of MDM2, p53 initiates its own degradation, which maintains a short half-life and low levels of the p53 protein in the absence of stress [8].

*Mdm2* knockout in mice leads to early embryonic lethality, which is rescued by the additional deletion of *p53*, demonstrating that the key role of MDM2 is to antagonize the function of p53 [13,14]. Aberrant upregulation of MDM2 expression has been observed in many sarcomas and other cancers, resulting in a decrease in the protein level and activity of p53 [9,10]. The main mechanisms of this MDM2 activation include amplification of the *MDM2* gene and genetic variants in its two promoters P1 and P2 [9,15]. *MDM2* amplification and overexpression occurs mostly in a mutually exclusive manner with *TP53* mutation, indicating that it may be one important of presumably several mechanisms of aberrant inactivation of p53 function in *TP53* wildtype tumors [8,9,16]. Like *MDM2* amplification, albeit less pronounced, the G-allele of SNP309 (rs2279744) in *MDM2* promoter P2 was also found associated with increased *MDM2* expression and a reduced rate of *TP53* mutation [17,18,19,20,21,22,23]. Moreover, most studies in Asian, but not in Caucasian populations found an association of the G-allele of SNP309 with an increased cancer risk [24,25,26].

**Figure 1:**
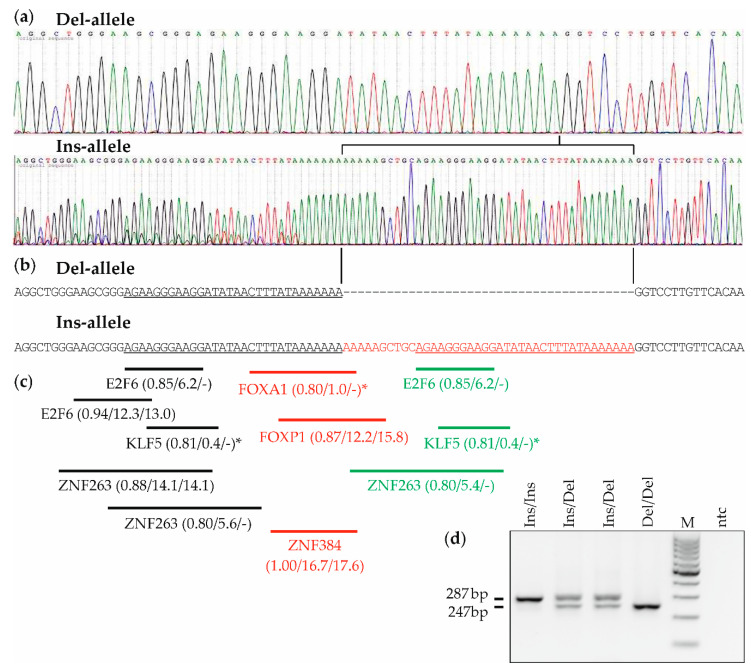
Analysis of the sequence context of *MDM2* (human homolog of mouse double minute 2) Indel polymorphism rs150550023. (**a**) Sequence chromatogram of the relevant genomic region of one representative patient each with the Del/Del and Ins/Ins genotype. (**b**) Sequence alignment of the Del- and the Ins-allele. The 40-bp insertion in the Ins-allele is highlighted in red. Note that an identical 30bp sequence out of these 40bp is also present in the Del-allele (underlined). (**c**) Predicted transcription factor binding sites in the rs150550023 Ins-allele (indicated by horizontal lines). Transcription factors binding exclusively to the Ins-allele but not the Del-allele are highlighted in red; extra binding sites within the Ins-allele of transcription factors that bind also to the Del-allele are highlighted in green. Numbers in parentheses indicate relative binding scores in JASPAR analysis/binding scores in JASPAR analysis/binding scores in CIS-BP analysis. * indicates binding to the (-) strand. (**d**) Genotyping by PCR and agarose gel electrophoresis of four representative samples with the indicated genotypes. The size of PCR-products derived from the Ins-allele (287bp) and the Del-allele (247bp) is indicated. M, DNA length marker, a 100-bp ladder with the 500-bp band present at higher concentration. ntc, nontemplate control (negative control).

Other studies have indicated an additional potential role in *MDM2* expression and cancer risk for an Indel (insertion/deletion) polymorphism located at position -2026_-2025 in the constitutive promoter P1 region of the *MDM2* gene [15,27,28,29]. Its Ins-allele is characterized by the insertion of the 40bp sequence (A)_5_GCTGCA(GAAGG)_2_ATATAACTTTAT(A)_7_, whereas the Del-allele has also one copy (and the Ins-allele hence two copies) of the 3′ 30bp of that 40bp sequence (Figure 1). This Indel variant was formerly termed rs3730485 or del1518, but the current official nomenclature according to NCBI Reference SNP (rs) database build 154 is rs150550023, which will be used hereafter. Compared to the extensively studied SNP309 in *MDM2* promoter P2, only a few studies have analyzed rs150550023. One study has used reporter assays in HeLa, HepG2, and JEG-3 human cell lines to indicate a role of rs150550023 in promoter P1 activity [15]. All tumor entities combined, fewer than 20 original studies and three meta-analyses have investigated the association of rs150550023 with cancer risk [27,28,29,30,31,32,33,34,35,36,37,38,39,40,41,42,43,44,45,46,47]. Most of these studies did not find an association, except in specific patient subpopulations, whereas associations with indicators other than cancer risk have hardly been studied for rs150550023. Four studies of breast cancer were reported, two in European and two in Asian populations [30,31,32,33]. Two studies reported a considerably increased breast cancer risk associated with the minor Del/Del, but not the Ins/Del genotype [32,33], whereas neither the other two, larger studies nor the meta-analyses found any association of rs150550023 with breast cancer risk [27,29,30,31]. Several studies have shown a strong linkage disequilibrium between rs150550023 and SNP309 [30,35,41], and one of these studies reported results that indicate a possible synergism between the minor alleles of these two polymorphisms [30].

SNP309 (rs2279744) is situated in promoter P2 in intron 1 of *MDM2*, and was shown to affect binding of transcription factor SP1, MDM2 expression, the risk, age at onset, and prognosis of human cancer, as well as p53 levels and the rate of *TP53* mutation [17,18,19,20,21,22,23,24,25,26]. The rationale of the present study is that rs150550023, which is located in the constitutive promoter P1 proximal to exon1 of *MDM2*, might have similar biological effects as SNP309. However, most of these potential biological roles of rs150550023 are still poorly characterized, and the main aim of the present study was to venture into some of the “white areas on the rs150550023 map”. We analyzed the association of rs150550023 with breast cancer risk, including clinically and biologically relevant subpopulations, with the age at onset, overall and metastasis-free survival, and potential synergism with SNP309. Our main focus was on expression analysis of MDM2, p53, and the p53 target genes p21, BAX, and PERP, which was indeed found associated with rs150550023 genotype. 

## 2. Results

### 2.1. Sequence Analysis and Genotyping of the MDM2 Indel Variant rs150550023

The sequence of human chromosome 12 containing the *MDM2* gene was retrieved from NCBI variation viewer assembly GRCh38.p12 (which contains the major Ins-allele), and the sequence surrounding rs150550023 was visualized in Figure 1b. We confirmed the sequence in our study population by Sanger sequencing of two randomly selected subjects each with the Ins/Ins and the Del/Del genotype (Figure 1a). The Ins-allele has an insertion of the 40bp sequence (A)_5_GCTGCA(GAAGG)_2_ATATAACTTTAT(A)_7_. A 30bp sequence identical to the 3′ part of this 40bp insertion is also present in the Del-allele, and is hence duplicated in the Ins-allele (Figure 1). Only the 10bp at the 5′ end of the 40bp insertion are unique to the Ins-allele (Figure 1). We note that due to this duplication of 30bp, it is a matter of opinion how to define the last nucleotide of the 40-bp insertion, which can be any of the 30 duplicated nucleotides. Indeed, the insertion was defined as A(GAAGG)_2_ATATAACTTTAT(A)_12_GCTGC in the now outdated nomenclature rs3730485, a shift by 30 nucleotides compared to the current nomenclature rs150550023.

We next performed in silico analyses of putative transcription factor binding sites around the rs150550023 site, applying JASPAR, CIS-BP, and The Human Protein Atlas databases [48,49,50]. We found predicted binding sites for FOXA1, FOXP1 (Forkhead Box Proteins A1 and P1) [51,52], and ZNF384 (Zinc Finger Protein 384) [53] unique to the Ins-allele, and binding sites for E2F6 (E2F Transcription Factor 6) [54], KLF5 (Krueppel-like Factor 5) [55], and ZNF263 (Zinc Finger Protein 263) [56] both in the Ins- and the Del-allele, but with one extra binding site each in the Ins-allele (Figure 1c). The highest binding scores could be confirmed with CIS-BP, an independent analysis tool [50]. A previous JASPAR analysis has focused on the breakpoint of the Del-allele (AAAAAAA/GGTCCTT) [30], which, however, is also present in the Ins-allele (Figure 1a,b). In fact, due to the 30-bp duplication there is no sequence that is unique to the Del-allele and absent from the Ins-allele; only sequence unique to the Ins-allele exists (Figure 1a,b). Genotyping was done by PCR and agarose gel electrophoresis. Representative examples of the obtained PCR bands of all three genotypes are shown in Figure 1d.

### 2.2. Distribution of rs150550023 Genotypes

rs150550023 was genotyped in a Central European (Austrian) hospital-based case-control study of 407 breast cancer patients and 254 control subjects. Table S1 shows the clinical and histopathological characteristics as well as the frequency of the rs150550023 genotypes within the study population. The control population (*p* = 0.72) and the breast cancer patient population (*p* = 0.60) were both in Hardy–Weinberg equilibrium for rs150550023. The frequency of the minor Del-allele of rs150550023 (MAF) was 0.399 in breast cancer patients and 0.429 in controls, close to the 0.416 MAF reported for Europeans by the GnomAD Project population database [57]. We had previously analyzed *MDM2* SNP309 (rs2279744) in our study population [21], and found that it was in a strong linkage disequilibrium with rs150550023 (D’ = 0.925; *p* < 0.0001), consistent with previous reports [30,35,41]. Accordingly, the rs150550023 Del-allele was found almost exclusively in a distinct SNP309T/rs150550023-Del haplotype. There were only 10 exceptions in our study population of 659 breast cancer cases and controls, in whom an rs150550023 Del-allele was linked to a SNP309 G-allele (Table S1).

### 2.3. MDM2 rs150550023 and Breast Cancer Risk

Determination of crude and adjusted odds ratios (OR), 95% confidence intervals (c.i.), and *p*-values revealed no significant associations of rs150550023 genotypes or alleles with breast cancer risk (Table 1), consistent with previous breast cancer studies and meta-analyses [27,29,30,31]. A previous study of lung, prostate, breast, and colon cancer has found higher odds ratios associated with the rs150550023 Del-allele in subjects with the SNP309TG genotype than with the SNP309TT genotype [30]. These findings indicate a possible synergism between the rs150550023 Del-allele and the SNP309 G-allele, even though a significantly increased risk was only found for prostate cancer [30,38].

Accordingly, we next determined ORs and confidence intervals in SNP309TT and SNP309TG subjects, as well as ORs and confidence intervals adjusted for SNP309 and age (Table 1 and Table 2). Due to the strong linkage disequilibrium between rs150550023 and SNP309, SNP309GG subjects are almost exclusively of the rs150550023 Ins/Ins genotype (see Section 2.2), precluding an analogous analysis in this subpopulation. Consistent with a previous breast cancer study [30], we found considerably, but non-significantly elevated odds ratios associated with the Del-allele in SNP309TG subjects compared to SNP309TT subjects (Table 2).

### 2.4. Exploratory Analysis of MDM2 rs150550023 and Breast Cancer Risk in Subpopulations

**Figure 2:**
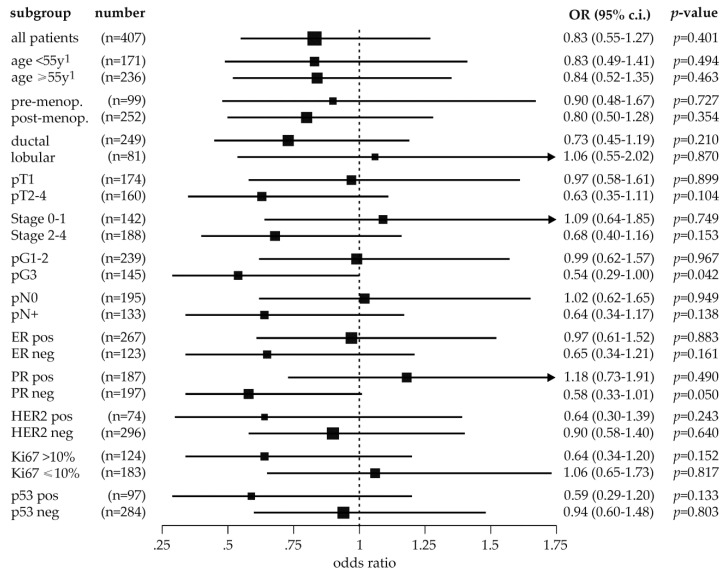
Forest plot illustrating the association of *MDM2* polymorphism rs150550023 with breast cancer risk in the indicated clinical and histopathological patient subpopulations in the recessive genetic model (Del/Del vs. Ins/Ins + Ins/Del). Squares indicate the odds ratios, their expanse the size of the subpopulations (also shown to the left), and horizontal lines the 95% confidence intervals. Odds ratios (OR), 95% confidence intervals (95% c.i.), and *p*-values (*p*) are in addition shown to the right. menop, menopausal; ER, estrogen receptor; PR, progesterone receptor; pos, positive; neg, negative; ^1^ patients aged under 55 years or ≥55 years at diagnosis were compared to control subjects of any age.

The potential association of rs150550023 with breast cancer risk in clinically and histo-pathologically relevant subpopulations has not been thoroughly investigated so far [30,31,32,33]. In contrast, associations with the age at onset, estrogen receptor (ER) status, p53 status, and Ki67-status have been demonstrated for SNP309 [17,18,21,58,59,60]. Accordingly, we explored whether associations of rs150550023 with breast cancer risk in these and other clinically relevant subpopulations exist, and determined ORs in the recessive genetic model (Del/Del vs. Ins/Ins + Ins/Del). This analysis revealed considerable differences in the odds ratios in subgroups stratified by tumor size (pT1 vs. pT2-4), stage (0–1 vs. 2–4), grade (pG1–2 vs. pG3), progesterone receptor (PR) status, Ki67 status (≤10% vs. >10% Ki67-positive tumor cells), and p53 status (Figure 2). Moreover, odds ratios tended to be lower in subgroups associated with advanced disease and a poor prognosis, such as pT2–4, stage 2–4, pG3 tumors with a negative estrogen and progesterone status and a positive HER2 and p53 status (Figure 2). However, only two of these associations were marginally significant: in high grade (pG3) tumors (OR, 0.54; 95% c.i., 0.29–1.00; *p* = 0.042), and in PR negative tumors (OR, 0.58; 95% c.i., 0.33–1.01; *p* < 0.05; Figure 2).

### 2.5. Association of MDM2 rs150550023 with the Age at Breast Cancer Onset

**Figure 3:**
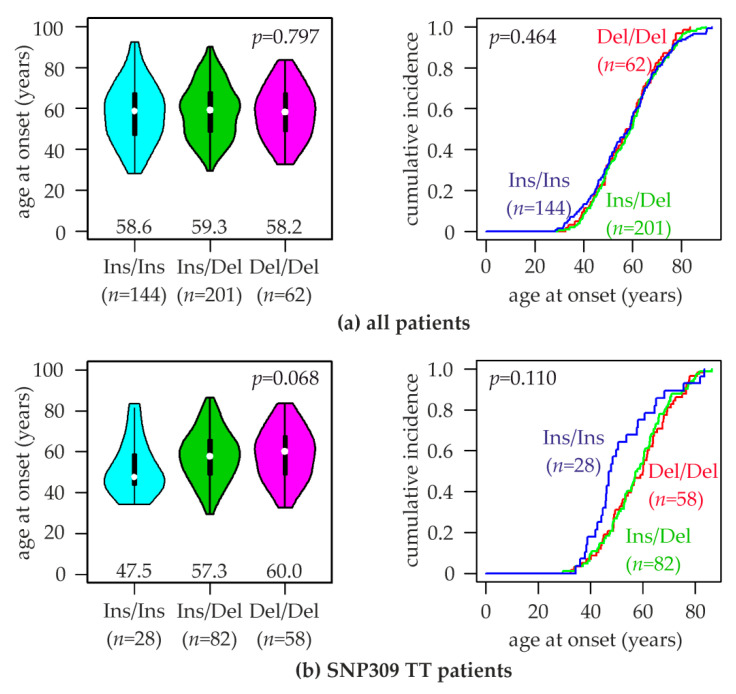
Association of rs150550023 genotypes with the age at breast cancer onset. Violin plots (left) and curves of the cumulative breast cancer incidence (right) of the indicated age at onset are shown for (**a**) all patients of the study population, and (**b**) patients with the SNP309TT genotype. rs150550023 genotypes (Ins/Ins, Ins/Del, Del/Del) and numbers of patients (*n*) are indicated. Numbers in left panels represent the median age at breast cancer onset of each genotype (indicated by white dots). *p*-values (*p*) in left panels were calculated with ANOVA, and in right panels with log-rank tests.

*MDM2* SNP309 was found associated with the onset age of breast cancer in several studies [18,21,58,60]. Since SNP309 is in strong linkage disequilibrium with rs150550023, its association with the age at onset was also investigated. We found the following mean ages of breast cancer onset for patients with the three rs150550023 genotypes: Ins/Ins, 57.7 ± 14.7 years (median, 58.6); Ins/Del, 58.6 ± 13.1 years (median, 59.3); Del/Del, 57.7 ± 12.8 years (median, 58.2; *p*~0.8, ANOVA; Figure 3a).

In carriers of the SNP309TT genotype, this analysis revealed pronounced differences in the age at onset: Ins/Ins, 52.1 ± 13.3 years (median, 47.5); Ins/Del, 57.9 ± 12.4 years (median, 57.6); Del/Del, 58.5 ± 12.7 years (median, 60.0; *p*~0.07, ANOVA; Figure 3b). Thus, patients with the Ins/Ins genotype exhibited an 11.4 years younger median age at breast cancer onset than carriers of the Del-allele (47.5 vs. 58.9 years; *p* = 0.032, unpaired two-sided *t*-test) in the SNP309TT subpopulation. The curve of cumulative breast cancer incidence of Ins/Ins patients appeared biphasic, with a considerably steeper slope than Del-allele carriers up to an age of approximately 51 years, and a gentler incline thereafter (Figure 3b). This age roughly coincides with menopause. This biphasic increase in the cumulative incidence in Ins/Ins-SNP309TT patients also results in the median being off-center in the violin plots, and being considerably smaller than the mean (Figure 3b).

### 2.6. MDM2 rs150550023 and mRNA Levels of MDM2, p53, and p53 Target Genes

**Figure 4:**
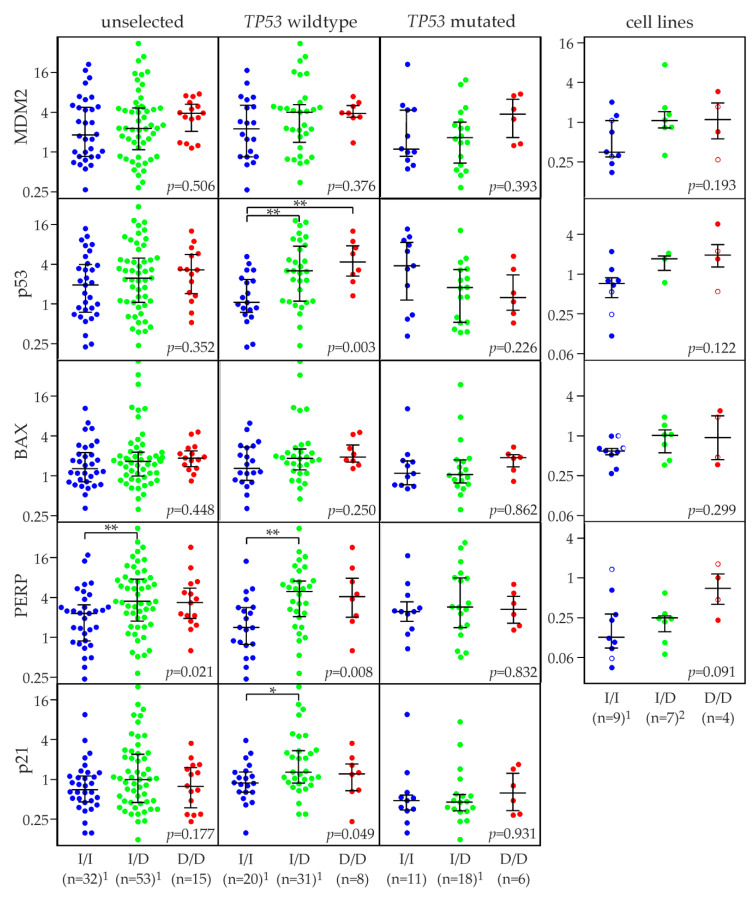
Association of rs150550023 genotypes with the expression of p53 target genes in human breast cancer. Strip charts of MDM2, p53, BAX, PERP, and p21 relative mRNA levels of breast tumors and cell lines, normalized to ß-actin. Results for unselected patients, patients with wildtype *TP53*, patients with mutated *TP53*, and breast cancer plus untransformed mammary epithelial cell lines (the latter indicated with open circles) are shown. Genotypes (Ins/Ins [I/I], Ins/Del [I/D], Del/Del [D/D]) and numbers of patients (n) are indicated. Horizontal lines indicate the first, second (i.e., median), and third quartiles. All *p*-values (*p*) indicated were calculated with ANOVA. Note that y-axes are logarithmic (log [2]). ^1^ The number of patients and cell lines is 1–2 lower than indicated in some of the panels due to unsuccessful qRT-PCR in some samples. ^2^ Expression of p53 was successfully determined in three instead of seven cell lines with genotype Ins/Del. * *p* < 0.05; ** *p* < 0.01 in comparisons of two groups; unpaired, two-sided *t*-tests.

In silico analysis has indicated differential binding of transcriptional repressors and activators to the Ins vs. Del alleles of rs150550023, which is located in the constitutive promoter P1 of *MDM2* (see Section 2.1). Accordingly, we applied qRT-PCR to quantify the expression of MDM2 mRNA in ≈100 human breast tumor samples as well as in 16 breast cancer and four untransformed mammary epithelial cell lines with predetermined rs150550023 genotypes (Figure 4). The expression of p53 itself, and of the p53 target genes BAX [61], PERP [62], and p21 (CDKN1A) [63] was analyzed in parallel. As expected, the mean expression of p53 target genes was higher in wildtype *TP53* tumors compared to tumors with a mutated *TP53* (as determined by sequencing; Appendix A): MDM2, 1.5-fold higher (*p =* 0.07; unpaired, two-sided *t*-test); BAX, 1.4-fold higher (*p =* 0.08); p21, 2.3-fold higher (*p =* 0.0001). Thus, p53 status had a comparably modest effect on BAX expression, consistent with findings in p53 null hematopoietic cells [7,64]. Although PERP expression can be potently induced by p53 [65], we found virtually no effect of p53 status on steady-state PERP mRNA levels (0.94-fold change; *p =* 0.77), as reported previously [66]. Mean mRNA levels of p53 itself were 1.23-fold elevated in *TP53* wildtype tumors (*p =* 0.4), consistent with the finding that p53 can bind to and activate its own promoter [64,67].

*MDM2* is a p53 target gene and engaged in a negative regulatory feedback loop with p53. Thus, we expected a more direct, proportionally larger impact of rs150550023 genotype on MDM2 expression in tumors with a mutated *TP53* gene, in which the additional, potentially confounding effects of p53 on MDM2 expression are absent. In the *TP53* wildtype background, MDM2 expression in Ins/Del tumors was higher than in Ins/Ins tumors, but comparable to Del/Del tumors (Ins/Del + Del/Del vs. Ins/Ins tumors: 1.5-fold up, *p =* 0.18; Figure 4). In the *TP53* mutated background, MDM2 expression in Ins/Del tumors was also higher than in Ins/Ins tumors, but was further elevated in Del/Del tumors (2-fold; *p =* 0.12). In contrast to *MDM2*, any effect of rs150550023 genotype on other p53 target genes is expected to be indirect, via p53. Indeed, BAX, PERP, and p21 expression was virtually unaffected by rs150550023 genotype in tumors with mutated *TP53* (*p =* 0.862, *p =* 0.832 and *p =* 0.931, respectively; ANOVA; Figure 4). In *TP53* wildtype tumors, BAX, PERP, and p21 roughly reproduced the expression pattern of MDM2: approximately equal expression levels in Ins/Del and Del/Del tumors, which were both higher than those in Ins/Ins tumors (Figure 4). The following relative expression levels of Ins/Del + Del/Del vs. Ins/Ins tumors were observed: BAX, 1.5-fold higher (*p =* 0.07); PERP, 2.7-fold higher (*p =* 0.002); p21, 1.8-fold higher (*p =* 0.02). Interestingly, p53 mRNA levels associated with the Ins/Del genotype were also higher compared to Ins/Ins (2.4-fold, *p =* 0.004), and were further elevated in the Del/Del genotype (1.5-fold, *p =* 0.04) in *TP53* wildtype, but not *TP53* mutant tumors (Figure 4). The expression patterns in cell lines were very similar to those in *TP53* wildtype tumors for all genes analyzed (Figure 4).

**Figure 5:**
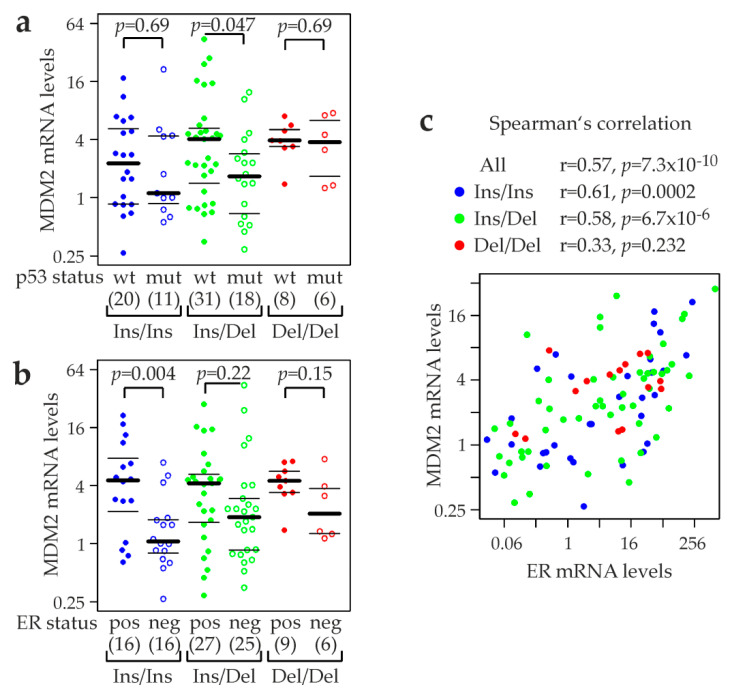
Association of rs150550023 genotypes with MDM2 mRNA expression, p53 status, and ER status in human primary breast tumors. rs150550023 genotypes and numbers of patients are indicated underneath each group. Relative MDM2 and ER (ESR1; estrogen receptor) mRNA levels, normalized to ß-actin, are shown on a logarithmic (log[2]) scale. (**a**) MDM2 mRNA expression in six groups of breast tumors stratified by rs150550023 genotype and p53 status (wt, wildtype; filled circles, and mut, mutated; open circles). (**b**) MDM2 mRNA expression in six groups of breast tumors stratified by rs150550023 genotype and ER status (pos, positive; filled circles, and neg, negative; open circles). (**c**) Correlation between MDM2 and ESR1 (ER) mRNA expression. Spearman’s rho (rank correlation coefficients) and corresponding *p*-values are indicated for all patients, and separately for each rs150550023 genotype (visualized by the indicated color code). (**a**,**b**) *p*-values (*p*) indicated were determined with unpaired, two-sided *t*-tests. Horizontal lines indicate the first, second (i.e., median), and third quartiles.

Next, we analyzed the combined effects on MDM2 mRNA expression of rs150550023 genotype and p53 status as well as estrogen receptor (ER) status (Figure 5). Mean and median MDM2 levels of tumors with a mutated *TP53* were clearly lower than in *TP53* wildtype tumors in patients with the Ins/Ins genotype (mean, 1.2× lower; median, 2× lower; *p =* 0.69; unpaired, two-sided *t*-test) and the Ins/Del genotype (mean, 2× lower; median, 2.4× lower; *p =* 0.047), but were only slightly reduced in patients with the Del/Del genotype (mean, 1.16× lower; median, 1.04× lower; *p =* 0.69; Figure 5a).

Like p53, the estrogen receptor (ER) binds to and activates *MDM2* promoter P2 [59,68,69], and we found a similar expression pattern in ER positive vs. negative tumors. Ins/Ins: mean, 3× lower in ER neg than ER pos tumors; median, 4.3× lower; *p =* 0.004; Ins/Del: mean, 1.5× lower; median, 2.2× lower; *p =* 0.22; Del/Del: mean, 1.7× lower; median, 2.2× lower; *p =* 0.15 (Figure 5b). From a different perspective, rs150550023 genotype had an impact on MDM2 expression only in ER negative tumors, whereas MDM2 levels of the three genotypes were very similar in ER positive tumors (Figure 5b). Similarly, the effect of rs150550023 genotype on MDM2 expression was much stronger in tumors with a mutated *TP53* than in *TP53* wildtype tumors (Figure 5a). We further analyzed the correlation of MDM2 and ESR1 (ER) mRNA levels using Spearman’s rank correlation and scatterplots for visualization (Figure 5c). A strong positive correlation was observed in the study population of 110 tumors (ρ *=* 0.57; *p =* 7 × 10^−10^). The more Ins-alleles that were present, the stronger the correlation observed, as demonstrated by higher values obtained for Spearman’s rho (ρ): Ins/Ins, ρ *=* 0.61, *p =* 0.0002; Ins/Del, ρ *=* 0.58, *p =* 7 × 10^−6^; Del/Del, ρ *=* 0.33; *p =* 0.23 (Figure 5c).

### 2.7. Association of MDM2 rs150550023 with Expression of p53 Protein

**Figure 6:**
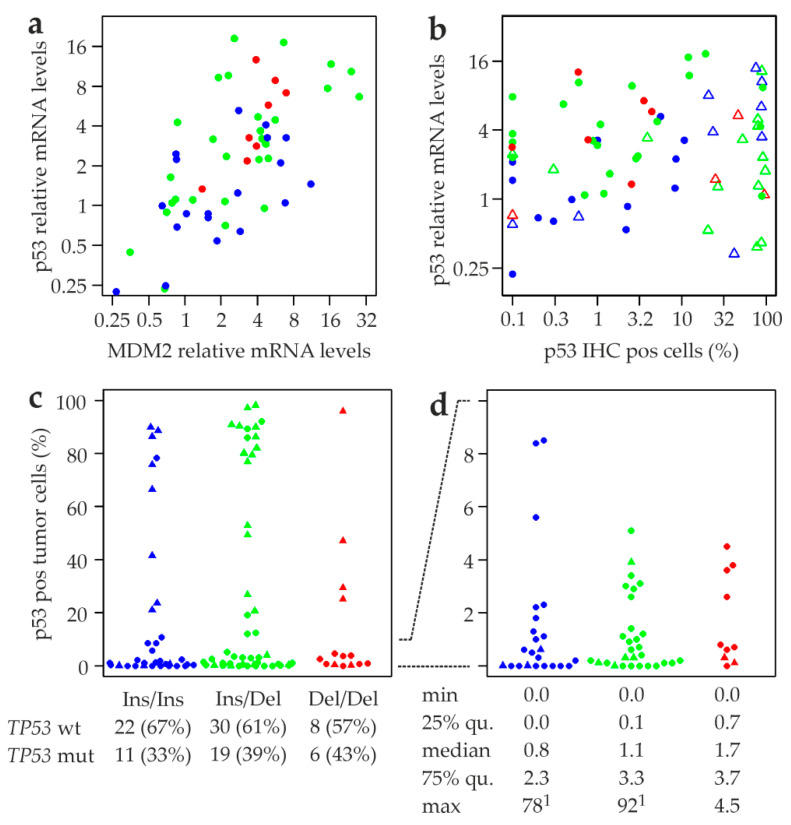
Association of rs150550023 genotypes with p53 protein in human primary breast tumors. (**a**) Correlation between MDM2 and p53 mRNA expression. (**b**) Correlation between p53 mRNA and protein expression. p53 positive tumor cells (%) were determined by immune histochemistry in breast tumors with the indicated rs150550023 genotype and *TP53* status. (**c**) p53 positive tumor cells (%) in breast tumors with the three indicated rs150550023 genotypes and *TP53* status. The numbers and frequencies (%) of the patients with a wildtype and mutated p53 are indicated underneath each group. (**d**) The part of panel (c) with 0-10% p53 positive tumor cells is shown. Numbers underneath indicate the minimum, median, maximum, 25% and 75% quantiles (qu.) of p53 positive tumor cells (%) for each rs150550023 genotype, considering only tumors with a wildtype *TP53*. rs150550023 genotypes are color coded as follows: Ins/Ins, blue; Ins/Del, green; Del/Del, red. *TP53* status is indicated as follows: wildtype (wt), circles; mutated (mut), triangles. ^1^ represented in panel (c) only.

We next found a strong positive correlation between MDM2 and p53 mRNA in *TP53* wildtype tumors (Spearman’s rank correlation coefficient ρ *=* 0.65, *p =* 6 × 10^−8^; Figure 6a), but a much weaker one in tumors with mutated *TP53* (ρ *=* 0.4, *p =* 0.02; data not shown). Within the *TP53* wildtype subtype, the weakest correlation was found in Ins/Ins tumors (ρ *=* 0.5, *p =* 0.03), compared to Ins/Del (ρ *=* 0.66, *p =* 9 × 10^−5^) and Del/Del tumors (ρ *=* 0.69, *p =* 0.07; Figure 6a). On average, Del/Del tumors had higher levels of both MDM2 and p53 mRNA than Ins/Ins tumors. Specifically, the p53 mRNA levels of half of the Del/Del tumors were higher than the maximum level of Ins/Ins tumors (Figure 6a; see also Figure 4). Moreover, the range of mRNA levels of both MDM2 and p53 was considerably narrower in Del/Del than in Ins/Ins and Ins/Del tumors. For example, the difference between the highest and lowest MDM2 levels was 41-fold in Ins/Ins tumors, but only 5-fold in Del/Del tumors (Figure 6a). We next analyzed the correlation of p53 mRNA and protein as a function of rs150550023 genotype (Figure 6b). Immune histochemistry (IHC) revealed that most tumors with a mutated *TP53* gene exhibited well above 20% p53 positive cells (Figure 6b,c), most likely because those mutant p53 proteins have lost the ability to activate MDM2 and other regulatory feedback loops (Appendix A).

The rate of *TP53* mutation was non-significantly elevated in Del/Del tumors, which was, however, not confirmed by p53 IHC status of the complete study population (Figure 6c; Table S1). Since p53 protein levels in tumors with a mutated *TP53* are thus not the result of physiological regulation, we focused our analysis on *TP53* wildtype tumors, in which we found a marginally significant positive correlation between p53 mRNA levels and p53 IHC positive cells (ρ *=* 0.3, *p =* 0.05; Figure 6b). This correlation was strongest in Ins/Ins tumors (ρ *=* 0.46, *p =* 0.1), weaker in Ins/Del tumors (ρ *=* 0.19, *p =* 0.4; Figure 6b), and weakest in Del/Del tumors (ρ *=* 0.14, *p =* 0.8; Figure 6b). In this *TP53* wildtype subpopulation, we found that Del/Del tumors exhibit considerably higher average p53 mRNA levels than Ins/Ins tumors, but without a correspondingly higher number of p53 protein positive cells (Figure 6b). Thus, Del/Del tumors appear to require higher levels of p53 mRNA to attain comparable levels of p53 protein. Like p53 and MDM2 mRNA levels, the number of p53 positive cells was in a narrower range in Del/Del tumors compared to Ins/Ins and Ins/Del tumors (Figure 6b–d). *TP53* wildtype Del/Del tumors had a maximum of 4.5% p53 positive cells; all tumors with a higher fraction had a mutated *TP53* (Figure 6c,d). Whereas the majority of Ins/Ins and Ins/Del tumors with >10% p53 positive cells also had a mutated *TP53* gene, we observed 2/22 tumors with wildtype *TP53* and >10% p53 positive cells in Ins/Ins tumors (maximum, 78%), and 6/30 Ins/Del tumors (maximum, 92%; Figure 6c,d). On the other hand, we also found a large fraction of tumors with the Ins/Ins and Ins/Del genotype with no or almost no p53 positive cells, which was not observed in Del/Del tumors (Figure 6d). In spite of the lower maximum of p53 positive cells in Del/Del tumors, the median, 25% and 75% quantiles were actually highest in Del/Del tumors and lowest in Ins/Ins tumors (Figure 6d).

### 2.8. Association of MDM2 rs150550023 with Breast Cancer Prognosis

The overall survival (OS) and metastasis-free survival (MFS) of the three *MDM2* rs150550023 genotypes was compared in univariable and multivariable Cox proportional hazards analyses of 134 patients treated between 1991 and 1994, for whom detailed follow-up records were available (Table 3 and Table 4). The status of p53, ER, and PR as well as SNP309 genotype were included as additional variables in this analysis. No evidence for an association of rs150550023 genotype with breast cancer prognosis was found in any of these analyses. Curiously, Kaplan–Meier analyses revealed a significant association of the Ins/Ins genotype with a poor survival free of metastases to distant lymph nodes in patients with the SNP309TT genotype (*p =* 0.01), with ER negative tumors (*p =* 0.006), and with a mutated *TP53* (*p =* 0.03; data not shown). In contrast, no association was found in the analyses of the survival free of metastases to the liver, lung, bone, brain, and pleura (data not shown).

## 3. Discussion

Here we report a comprehensive analysis of the rs150550023 Indel polymorphism located in the constitutive promoter P1 of the human *MDM2* gene, including the first investigation of rs150550023 association with the age at onset and prognosis of any cancer, and with the mRNA expression of MDM2 and other relevant genes in tumor tissue. We did not find an association of rs150550023 with breast cancer risk, in agreement with most previous studies and meta-analyses [27,28,29,30,31,32,33,34,35,36,37,38,39,40,41,42,43,44,45,46,47]. The reports of a nonsignificantly elevated vs. a nonsignificantly reduced risk associated with the Del-allele (like in the present study) are divided roughly equally. However, in those few studies reporting a significant association, the Del-allele was associated with an increased risk [30,32,33,39]. On the other hand, a significantly reduced risk associated with the Del-allele was found in carriers of the SNP309TT genotype for endometrial cancer in the recessive and dominant model [38]. This modifier effect of SNP309 upon the cancer risk associated with rs150550023 was significant only in endometrial cancer, but a nonsignificant trend was also observed in ovarian cancer, colon cancer, lung cancer in females, and breast cancer, in agreement with the present study [30,38]. In all cases, odds ratios associated with the Del-allele were considerably lower in carriers of the SNP309TT genotype than in carriers of the SNP309TG genotype. These two subpopulations differ with respect to the presence of the SNP309 G-allele, indicating a functional synergism between the SNP309 G-allele and the rs150550023 Del-allele (and/or vice versa, between the SNP309 T-allele and the rs150550023 Ins-allele) [30,38]. In addition to cancer risk, this potential synergism manifested in the age of breast cancer onset in the present study, which was ≈10 years earlier in patients with the Ins/Ins genotype than in carriers of the Del-allele in the SNP309TT subpopulation, but not in unselected patients. 

We also determined ORs in the recessive genetic model in various clinically and molecularly relevant breast cancer subpopulations. This analysis revealed that odds ratios tended to be lower in subgroups associated with advanced disease and a poor prognosis, such as pT2–4, stage 2–4, pG3, ER and PR negative, as well as HER2 and p53 positive. Thus, the Del/Del genotype was less frequent in those subgroups of advanced disease than in the corresponding subgroups with a good prognosis (e.g., pT1, stage 0–1; Table S1; Figure 2), indicating that Del/Del patients are more likely than Ins-carriers to develop a less aggressive form of breast cancer. Nevertheless, no association of rs150550023 with breast cancer prognosis was detected in our survival analysis.

In a collection of ≈100 primary human breast tumors, we extensively analyzed the association of rs150550023 genotype with the mRNA levels of MDM2, p53, and three additional p53 target genes, as well as p53 protein expression and the mutual interdependencies of these expression levels. Our major findings are as follows: (i) mean and median MDM2 mRNA levels were ≈1.5–2-fold higher in Del/Del than Ins/Ins tumors, and this difference was more pronounced in tumors with a mutated *TP53* than in wildtype *TP53* tumors. This is likely due to confounding effects of wildtype p53, which also increases MDM2 levels, but independently of rs150550023. (ii) The mRNA levels of the p53 target genes BAX, PERP, and p21 are also elevated in Del/Del tumors. However, opposite to MDM2, this association with rs150550023 genotype was restricted to *TP53* wildtype tumors. Hence, it is most likely mediated by p53. (iii) Interestingly, p53 mRNA levels were also ≈3.5-fold elevated in Del/Del vs. Ins/Ins tumors with a wildtype *TP53*, but not with a mutated *TP53*. However, this upregulation of p53 mRNA is not accompanied by an upregulation of p53 protein. Thus, Del/Del tumors appear to require higher levels of p53 mRNA to attain comparable levels of p53 protein. (iv) The levels of MDM2 mRNA, p53 mRNA, and p53 protein in Del/Del tumors are in a narrower range than in Ins/Ins tumors. (v) A positive correlation of MDM2 and ER mRNA levels was found in Ins/Ins, but not in Del/Del tumors. Moreover, MDM2 levels were significantly higher in ER positive vs. negative tumors with the Ins/Ins, but not the Del/Del genotype, indicating that this key activator of the *MDM2* promoter P2 had a more modest effect on MDM2 expression in Del/Del tumors. (vi) Ins/Del tumors exhibited levels intermediate between Ins/Ins and Del/Del tumors for most parameters analyzed. Collectively, these findings indicate that the p53-MDM2 regulatory hub is strengthened and regulated more tightly in Del/Del tumors. This may make MDM2 expression levels more robust towards effects of other regulators such as the ER; and lead to a narrower, more tightly controlled range of the levels of MDM2 and p53. Of note, amplification of the *MDM2* gene typically leads to much more than twofold upregulation of MDM2 expression [9], and the compensatory mechanisms observed here are unlikely to be effective.

The higher levels of MDM2 mRNA in Del/Del tumors are consistent with our JASPAR analysis, which predicted extra binding sites in the Ins allele for the transcriptional repressors FOXP1, E2F6, ZNF263, and ZNF384 [52,53,54,56,70,71]. Although the transcriptional activators FOXA1 and KLF5 [51,55] also have predicted additional binding sites in the Ins allele, they both bind to the minus-strand of *MDM2* promoter P1 and exhibit rather modest JASPAR binding scores. Reporter assays upon transient transfection with a fragment of *MDM2* promoter P1 containing rs150550023 have shown a ≈2-fold higher promoter activity associated with the Ins-allele in HeLa, HepG2, and JEG-3 cell lines [15]. However, the effect attributed to rs150550023 in these reporter assays may actually be due to SNP rs937282 located ≈1000bp downstream of rs150550023 [15,34]. Moreover, this study used an experimental system quite different from our quantification of endogenous MDM2 mRNA in ≈100 human breast tumors, and included neither breast epithelial nor breast cancer cells, nor *MDM2* promoter P2 with its potential modifying effects on MDM2 expression. The consequences of perturbations of the MDM2-p53 signaling hub appear to be quite different in ectoderm-, mesoderm-, and endoderm-derived cell types [3,72], and analyses of additional experimental systems and cell/tissue types may well be essential to arrive at a more complete picture of the functional impact of the rs150550023 Indel polymorphism.

## 4. Materials and Methods

### 4.1. Study Population

This study was approved by the Institutional Review Board (IRB; “Ethikkommission”) of the Medical University of Vienna, Austria (MUV; protocol 141/2002), and is annually reviewed. Only women of Central European descent from the same geographical area were included in this study. Healthy females and consecutive patients with benign gynecological lesions without any malignancies (breast or other cancer) in their personal history were enrolled as nested controls between 2002 and 2004 at the Department of Obstetrics and Gynecology, MUV (*n* = 255). A total of 276 consecutive female breast cancer patients treated between 2002 and 2004, and another 134 consecutive patients treated between 1991 and 1994 at the Department of Obstetrics and Gynecology, MUV, were enrolled in this study. From the latter 134 patients, detailed follow-up records as well as fresh-frozen tumor tissue were available. Malignant breast cancer of all patients was confirmed by histopathology. Table S1 depicts the clinical and histopathological characteristics of the study population. Written informed consent was obtained from all participants enrolled between 2002 and 2004. The patients treated between 1991 and 1994 were enrolled retrospectively, and the IRB approved a waiver of specific informed consent for those patients. Three patients and one control subject were excluded from further analyses due to technical genotyping failure. Accordingly, all analyses shown are based on the 407 breast cancer patients and 254 controls for whom genotyping was successful.

### 4.2. Cell Lines

Untransformed HMEC (human mammary epithelial cells) were kindly provided by M. R. Stampfer and grown in MEGM medium [73]. All other cell lines were purchased from DSMZ (“Deutsche Sammlung von Mikro-Organismen und Zellkulturen”, Braunschweig, Germany): Cal51, HCC-1143, HCC-1937 and Kpl-1, or ATCC (American Type Culture Collection, Manassas, USA): AU565, BT474, Cama1, Hs578T, Hs578Bst, MCF7, MCF10A, MCF10F, MDA-MB-231, MDA-MB-435, MDA-MB-453, MDA-MB-468, SK-BR-3, T47D, and ZR75-1, and were cultured as described [74]. DSMZ and ATCC authenticate all cell lines by STR profiling and other methods before distribution. Genomic DNA and total RNA were isolated from all cell lines within three to eight passages after receipt as described [74,75].

### 4.3. In Silico Analyses

In silico analyses of putative transcription factor binding sites around the rs150550023 site were performed using the JASPAR database [48] available from http://jaspar.genereg.net/ with default settings. Accordingly, only transcription factors with a relative binding score of ≥0.8 were considered further. Those transcription factors were subjected to in silico expression analysis using The Human Protein Atlas [49] available from http://www.proteinatlas.org, and all transcription factors not expressed in breast tissue were omitted from further analysis. Finally, only those transcription factors with a differential binding between the Ins- vs. the Del-allele (i.e., a different number of binding sites and/or different relative binding scores) were included in the final result and visualized in Figure 1c. The JASPAR analysis was repeated with CIS-BP, an independent database available from http://cisbp.ccbr.utoronto.ca/ with default settings. i.e., a threshold of ≥8 for the binding score.

### 4.4. DNA Isolation and Genotyping

All samples were genotyped by using genomic DNA extracted from peripheral lymphocytes with the QIAamp DNA Blood Midi kit (Qiagen, Venlo, The Netherlands), and from fresh-frozen tumor tissue with the High Pure PCR Template Preparation Kit (Roche, Vienna, Austria) as described [66,76]. SNP rs150550023 (rs3730485; del1518) in promoter P1 of the *MDM2* gene was genotyped by conventional PCR. Reactions contained 40 ng of genomic DNA, RedTaq PCR reaction mix (Sigma-Aldrich, Vienna, Austria) and a primer pair described in [32] in a reaction volume of 25 µL. PCR products were analyzed by gel electrophoresis with 2% agarose gels. The Ins-allele gave rise to a 287bp PCR product, and the Del-allele to a 247bp PCR product [32]. The following quality control measures were followed: (i) ≈200 samples were genotyped in duplicate, including five samples from separate DNA isolations; (ii) 45 samples were re-genotyped with a different primer pair described in [15]; (iii) genotypes of four samples (one patient and one control each with genotypes Ins/Ins and Del/Del) were confirmed by Sanger sequencing of PCR-products, after purification with the M-pure kit (Analytik Jena, Vienna, Austria; Figure 1a). (iv) Sizes of PCR products with both primer pairs were verified for six samples with a Bioanalyzer 2100 and a DNA 1000 Nano LabChip kit (Agilent, St. Clara, CA, USA) to be exactly as predicted from the corresponding sequence retrieved from NCBI variation viewer assembly GRCh38.p12, demonstrating that PCR-products observed are specific; (v) laboratory staff was blinded regarding patient or control status and identity of duplicates; (vi) genotypes were independently scored by three authors based on agarose gel results, and results consolidated by consensus. Genotyping failed for three patients and one control due to poor quality of genomic DNA. All samples were previously genotyped for *MDM2* SNP309 (rs2279744) as described [21].

### 4.5. qRT-PCR Analysis of mRNA Expression Levels

Isolation of total RNA from 111 fresh-frozen tumor samples with TRIreagent (Sigma, Vienna, Austria), quality control of the isolated RNA with the Bioanalyzer 2100 (Agilent, St. Clara, USA), reverse transcription with the High-Capacity cDNA Archive Kit (Applied Biosystems, Brunn/Gebirge, Austria), and quantification of relative mRNA levels of *MDM2*, p53, *BAX*, *PERP,* and p21 has been described previously [66,77]. Briefly, each sample was analyzed in duplicate by a quantitative reverse transcription PCR (qRT-PCR; real-time PCR) with an Applied Biosystems 7500 fast instrument, using the following primers and gene-specific probes obtained from Applied Biosystems: *MDM2*, hs00234753_m1; p53, hs_001533340_m1; *BAX*, hs00414514_m1; *PERP*, hs00953482_g1; p21, hs00355782_m1; and β-actin (control), hs_99999903_m1. In each qRT-PCR run, two to four negative controls (2.5 μL ddH2O instead of cDNA) were included and run in parallel. No signal was obtained in any of these reactions. As a positive control, duplicate samples of serial dilutions of a cDNA standard (cultured normal breast epithelial cells; HMECs) were included in each run. The mRNA levels of *MDM2*, p53, *BAX*, *PERP,* and p21 were normalized to those of β-actin in each sample (producing ΔCt values), and were further normalized by expressing the levels of all tumor samples relative to those of four control samples of normal breast tissue (producing ΔΔCt values). Four non-cancer cell lines were used as normalization controls for the analyses in breast cancer cell lines (HMEC, Hs578Bst, MCF10A, and MCF10F; indicated as open circles in Figure 4). All relative mRNA expression levels are presented as 2^−ΔΔCt^ values (i.e., as linear values, but on a logarithmic ordinate) as described [66,77].

### 4.6. Statistical Analyses

Statistical analyses were performed with R 3.3.2, an open-source language and environment for statistical computing available from www.r-project.org [78]. Hardy–Weinberg equilibrium was evaluated by chi-square tests with Yates’ continuity correction. Confidence intervals and *p*-values associated with odds ratios were calculated by the mid-P exact method. We consider our subgroup analyses (Figure 2) as exploratory, and therefore we did not adjust for multiple testing, in agreement with previous recommendations [79]. *p*-values for the cumulative breast cancer incidence were determined by log-rank tests as described [80]. Differences with respect to age at onset, mRNA levels, and numbers (%) of p53 positive cells between two groups were analyzed with unpaired, two-sided *t*-tests, and between three groups with ANOVA. Correlations between expression levels of two genes were analyzed by determining Spearman’s rank correlation coefficients (rho/ρ/r) and corresponding *p*-values. Follow-up details of our study population have been described [66,77]. The overall and metastasis-free survival was analyzed by univariable and multivariable Cox proportional hazard models, and included the variables rs150550023 genotype, SNP309 genotype, *TP53* status, ER status, and PR status. The subcategories of these variables were coded as follows: rs150550023 genotype, Ins/Ins = 0, Ins/Del = 1, Del/Del = 2; SNP309 genotype, TT = 0, TG = 1, GG = 2; ER and PR status, positive = 0, negative = 1; *TP53* status, wildtype = 0, mutated = 1 (determined by sequencing; Appendix A). All *p*-values shown are two-sided. Associations with *p*-values <0.05 were considered statistically significant.

## 5. Conclusions

The results of our expression analyses are consistent with a model in which the p53-MDM2 regulatory hub is strengthened and regulated more tightly in Del/Del tumors, making it more robust towards effects by other regulators of this hub such as the ER; leading to a narrower, more tightly controlled range of the levels of MDM2 and p53. This new set-point of the p53-MDM2 regulatory loop with a higher basal activity in Del/Del is likely initiated by a higher transcriptional activity of MDM2 promoter P1 harboring the Del-allele. These higher MDM2 levels may then lead to a compensatory upregulation of p53 mRNA, likely executed by the multiple feedback loops which regulate p53 mRNA transcription, stability, and translation efficiency. Among others, these regulatory feedback loops involve binding of p53 to its own promoter, and binding of p53, MDM2, and the p53 target gene WIG1 to p53 mRNA [12,67,81]. The higher basal levels of both p53 and MDM2 mRNA presumably neutralize each other, leading to a higher rate of both synthesis and degradation of p53 protein in the absence of stress. This may explain why the higher levels of p53 mRNA do not lead to higher p53 protein levels, which is important since basal p53 protein levels must be neither too high nor too low. However, the higher basal p53 mRNA levels may set the stage for a more potent p53 stress response with a higher amplitude and longer sustainability [81]. Indeed, p53 mRNA levels may be a limiting factor for p53 protein levels in Ins/Ins, but not Del/Del tumors, indicated by a positive correlation in the former, but not the latter. The equally higher MDM2 levels in Del/Del become irrelevant in a stress response, since its negative regulation of p53 is rapidly inactivated, and MDM2 may even be turned into an activator of p53 [12]. However, we did not find clear phenotypic consequences of the proposed impact of rs150550023 genotype upon regulation of the p53-MDM2 hub with respect to cancer risk, age of onset, rate of *TP53* mutation, or survival.

## Figures and Tables

**Table 1:** Association of *MDM2* rs150550023 genotypes and alleles with breast cancer risk.

Genotypes/Alleles	Unadjusted			Adjusted for SNP309 and Age		
	OR	95% c.i.	*p*-Value	OR	95% c.i.	*p*-Value
Del/Del vs. Ins/Ins	0.78	0.48–1.24	0.289	0.68	0.36–1.27	0.198
Del/Del vs. Ins/Del	0.88	0.56–1.37	0.564	0.91	0.51–1.61	0.732
Del/Del vs. Ins/Del + Ins/Ins	0.83	0.55–1.27	0.401	0.78	0.46–1.34	0.371
Ins/Del vs. Ins/Ins	0.88	0.62–1.25	0.488	0.82	0.52–1.29	0.353
Ins/Del + Del/Del vs. Ins/Ins	0.86	0.61–1.19	0.356	0.80	0.51–1.23	0.304
Del vs. Ins	0.88	0.70–1.11	0.276	0.82	0.60–1.12	0.215

Analyses of all breast cancer cases vs. controls of the indicated genotypes or alleles are shown, unadjusted or adjusted for SNP309 genotype and age as indicated. OR, odds ratio; 95% c.i., 95% confidence interval.

**Table 2:** Association of *MDM2* rs150550023 genotypes and alleles with breast cancer risk in subpopulations according to SNP309 genotype.

Genotypes/Alleles	in SNP309TT Subjects			in SNP309TG Subjects		
	OR	95% c.i.	*p*-Value	OR	95% c.i.	*p*-Value
Del/Del vs. Ins/Ins	0.56	0.26–1.23	0.145	2.11 ^1^	0.21–21.0	0.501
Del/Del vs. Ins/Del	0.79	0.47–1.33	0.376	2.04 ^1^	0.21–20.0	0.524
Del/Del vs. Ins/Del + Ins/Ins	0.73	0.44–1.20	0.221	2.06 ^1^	0.21–20.0	0.513
Ins/Del vs. Ins/Ins	0.72	0.33–1.54	0.388	1.04	0.64–1.70	0.863
Ins/Del + Del/Del vs. Ins/Ins	0.65	0.31–1.33	0.227	1.06	0.65–1.72	0.821
Del vs. Ins	0.76	0.53–1.10	0.137	1.09	0.69–1.73	0.718

Analyses of breast cancer cases vs. controls of the indicated genotypes or alleles are shown. Analyses were performed in the subgroup of breast cancer patients and controls with the SNP309TT genotype, or with the SNP309TG genotype as indicated. OR, odds ratio; 95% c.i., 95% confidence interval. ^1^ Note that only three patients and one control have the genotype rs150550023 Del/Del–SNP309TG (see Section 2.2).

**Table 3:** Univariable and multivariable analyses of the overall survival using a Cox proportional hazards model.

Variable	Univariable			Multivariable		
	HR	95% c.i.	*p*-Value	HR	95% c.i.	*p*-Value
**rs150550023**	0.96	0.68–1.36	0.821	0.92	0.64–1.33	0.668
**SNP309**	1.05	0.76–1.46	0.768	1.03	0.71–1.49	0.874
**p53 status**	1.36	0.84–2.19	0.204	1.08	0.64–1.84	0.768
**ER status**	1.32	0.84–2.06	0.228	1.17	0.71–1.95	0.532
**PR status**	1.45	0.91–2.30	0.117	1.45	0.86–2.45	0.163

HR, hazard ratio; 95% c.i., 95% confidence intervals; ER, estrogen receptor. Subcategories of the indicated variables were coded as described in Methods.

**Table 4:** Univariable and multivariable analyses of the metastasis-free survival using a Cox proportional hazards model.

Variable	Univariable			Multivariable		
	HR	95% c.i.	*p*-Value	HR	95% c.i.	*p*-Value
**rs150550023**	1.10	0.75–1.62	0.624	0.96	0.64–1.45	0.859
**SNP309**	0.80	0.54–1.18	0.266	0.77	0.50–1.18	0.231
**p53 status**	1.55	0.93–2.60	0.094	1.12	0.64–1.98	0.689
**ER status**	1.41	0.87–2.30	0.167	1.17	0.68–2.02	0.565
**PR status**	1.65	0.98–2.77	0.057	1.65	0.91–3.00	0.097

HR, hazard ratio; 95% c.i., 95% confidence intervals; ER, estrogen receptor; PR, progesteron receptor. Subcategories of the indicated variables were coded as described in Methods.

## Supplementary Materials

The following are available online at https://www.mdpi.com/2072-6694/12/11/3363/s1: Table S1: Clinical characteristics and genotype frequencies of rs150550023 of the study population.

## Appendix A

P53 status was determined as follows (detailed results will be reported elsewhere): for the patients enrolled between 2002 and 2004, p53 status had been determined by immune histochemistry (IHC) as part of the routine clinical management in Austrian hospitals, and was retrieved from their clinical records. The presence of ≥10% p53-positive tumor cell nuclei was scored as “p53 positive” in this routine clinical diagnosis, in agreement with national and international guidelines. P53 positivity in IHC is considered as indication of an inactivating mutation of *TP53*, which leads to a defective p53-MDM2 negative feedback loop and hence stabilization and accumulation of the mutated p53 protein [61,82,83,84,85]. For the patients treated between 1991 and 1994, the determination of p53 status was not yet part of their routine clinical management. Accordingly, we determined the p53 status for 105 of these patients retrospectively following IHC procedures of current routine diagnosis. Tissue microarrays with triplicate tumor tissue samples of these patients [86] were stained with monoclonal anti-p53 antibody (clone DO-7, Dako/Agilent, St. Clara, USA) at a dilution of 1:800 for one hour. In addition, *TP53* status was determined by sequencing of genomic DNA isolated from fresh-frozen, native tumor tissue of 127 of the patients treated between 1991 and 1994. A total of 116 patients were analyzed by Sanger sequencing of *TP53* exon 4-9 from both directions as described [66]. In addition, the complete *TP53* coding sequence of 78 patients was analyzed by ultra-deep sequencing (average sequencing depth of the *TP53* gene, 15,494 ± 12,434) with the TruSight Tumor 26 (TS26) gene panel on a MySeq instrument (Illumina, San Diego, USA). In total, 67 of those patients had previously been analyzed by Sanger sequencing, and initial discordant results (*n* = 10, mostly with <15% variant reads) were resolved by re-evaluating the original Sanger sequence chromatograms, and by repeating the Sanger sequencing. Altogether, the *TP53* sequence of 127 patients was successfully ascertained, 49 by Sanger sequencing only, 11 with targeted next-generation sequencing only, and 67 with both methods. Sequence variants detected in the *TP53* coding sequence were scored as *TP53* mutations, with the exception of (i) germline polymorphisms such as R72P (rs1042522); (ii) silent (synonymous) point mutations; (iii) variants detected in <10% of the Illumina reads; (iv) variants scored as benign or likely benign according to the IARC TP53 database at https://p53.iarc.fr/ or according to ACMG/AMP consensus guidelines [87].
