# Supplementary material for: The 40bp Indel Polymorphism rs150550023 in the MDM2 Promoter is Associated with Intriguing Shifts in Gene Expression in the p53-MDM2 Regulatory Hub
Source: Cancers (Basel). 2020 Nov 13;12(11):3363. doi: 10.3390/cancers12113363 (PMC7697608; doi:10.3390/cancers12113363)
Supplement: Supplementary file 1 [file cancers-12-03363-s001.pdf]

cancers

MDPI

The 40bp Indel Polymorphism rs150550023 in the *MDM2* Promoter is Associated with Intriguing Shifts in Gene Expression in the p53-MDM2 Regulatory Hub

Heidi Miedl, Bianca Dietrich, Klaus Kaserer and Martin Schreiber

Table 1. Clinical characteristics and genotype frequencies of rs150550023 of the study population.

			rs150550023 genotype						
		Total	Ins/Ins		Ins/Del		Del/Del		*p*-value
All subjects		661	225	(34.0%)	329	(49.8%)	107	(16.2%)	
Patients		407	144	(35.4%)	201	(49.4%)	62	(15.2%)	
Controls		254	81	(31.9%)	128	(50.4%)	45	(17.7%)	
Control subpopulations									
SNP309 genotype	TT	105	12	(11.4%)	49	(46.7%)	44	(41.9%)	$3.4*10^{-25}$
	TG	121	43	(35.5%)	77	(63.6%)	1	(0.8%)	
	GG	28	26	(92.9%)	2	(7.1%)	0	(0.0%)	
Patient subpopulations									
SNP309 genotype	TT	168	28	(16.7%)	82	(48.8%)	58	(34.5%)	$2.7*10^{-36}$
	TG	178	61	(34.3%)	114	(64.0%)	3	(1.7%)	
	GG	59	55	(93.2%)	3	(5.1%)	1	(1.7%)	
Age (years)	<55	171	65	(38.0%)	80	(46.8%)	26	(15.2%)	0.612
	≥55	236	79	(33.5%)	121	(51.3%)	36	(15.3%)	
Menopausal status	pre	99	36	(36.4%)	47	(47.5%)	16	(16.2%)	0.875
	post	252	88	(34.9%)	127	(50.4%)	37	(14.7%)	
	na	56	20	(35.7%)	27	(48.2%)	9	(16.1%)	
Tumor type	ductal	249	88	(35.3%)	127	(51.0%)	34	(13.7%)	0.565
	lobular	81	27	(33.3%)	39	(48.1%)	15	(18.5%)	
	other, na	77	29	(37.7%)	35	(45.5%)	13	(16.9%)	
Tumor size	pT1	175	65	(37.1%)	80	(45.7%)	30	(17.1%)	0.220
	pT2–4	160	54	(33.8%)	87	(54.4%)	19	(11.9%)	
	other, na	73	26	(35.6%)	34	(46.6%)	13	(17.8%)	
Stage	0–1	142	55	(38.7%)	60	(42.3%)	27	(19.0%)	0.035
	2–4	188	58	(30.9%)	106	(56.4%)	24	(12.8%)	
	other, na	77	31	(40.3%)	35	(45.5%)	11	(14.3%)	
Grade	pG1–2	239	81	(33.9%)	116	(48.5%)	42	(17.6%)	0.145
	pG3	145	56	(38.6%)	74	(51.0%)	15	(10.3%)	
	na	23	7	(30.4%)	11	(47.8%)	5	(21.7%)	
Lymph node status	pN0	195	75	(38.5%)	85	(43.6%)	35	(17.9%)	0.009
	pN+	133	36	(27.1%)	81	(60.9%)	16	(12.0%)	
	na	68	33	(48.5%)	35	(51.5%)	0	(0.0%)	
ER status	pos	267	86	(32.2%)	135	(50.6%)	46	(17.2%)	0.235
	neg	123	49	(39.8%)	59	(48.0%)	15	(12.2%)	
	na	17	9	(52.9%)	7	(41.2%)	1	(5.9%)	
PR status	pos	187	60	(32.1%)	89	(47.6%)	38	(20.3%)	0.044
	neg	197	74	(37.6%)	101	(51.3%)	22	(11.2%)	
	na	23	10	(43.5%)	11	(47.8%)	2	(8.7%)	
HER2 status	pos	74	26	(35.1%)	39	(52.7%)	9	(12.2%)	0.684
	neg	296	101	(34.1%)	147	(49.7%)	48	(16.2%)	

	na	37	17	(45.9%)	15	(40.5%)	5	(13.5%)	
	pos	97	29	(29.9%)	57	(58.8%)	11	(11.3%)	
p53 status	neg	284	102	(35.9%)	134	(47.2%)	48	(16.9%)	0.126
	na	26	13	(50.0%)	10	(38.5%)	3	(11.5%)	
	≤10%	183	59	(32.2%)	90	(49.2%)	34	(18.6%)	
KI67 pos cells	>10%	124	51	(41.1%)	58	(46.8%)	15	(12.1%)	0.160
	na	99	34	(34.3%)	52	(52.5%)	13	(13.1%)	

Numbers of all patients and controls, as well as number of patients in the indicated subgroups are shown. Parenthesized numbers show the fraction of patients (%) with the indicated genotypes. P-values were calculated with Chi-square tests, not including na subjects. ER, estrogen receptor; PR, progesterone receptor; na, status not available.

Publisher's Note: MDPI stays neutral with regard to jurisdictional claims in published maps and institutional affiliations.

© 2020 by the authors. Licensee MDPI, Basel, Switzerland. This article is an open access article distributed under the terms and conditions of the Creative Commons Attribution (CC BY) license (http://creativecommons.org/licenses/by/4.0/).
